# Supplementary material for: Reconciling Mining with the Conservation of Cave Biodiversity: A Quantitative Baseline to Help Establish Conservation Priorities
Source: PLoS One. 2016 Dec 20;11(12):e0168348. doi: 10.1371/journal.pone.0168348 (PMC5173368; doi:10.1371/journal.pone.0168348)
Supplement: S1 Dataset — (ZIP) [file pone.0168348.s002.zip › Taxa/Serra Norte/SN_2013/N5S/Lista N5S-38.pdf]

## CAVIDADE N5S-0038

| Classe | Ordem | Fam/Outros | Gên/Outros | Espécie | S-U | U-S | Zon. |
|---|---|---|---|---|---|---|---|
| Arachnida | Acari | Opilioacaridae | | sp.1 | | X | E |
| Arachnida | Acari | Rhagidiidae | | sp.20 | X | | P |
| Arachnida | Amblypygi | Phrynidae | *Heterophrynus* | *longicornis* | 3 | 2 | P |
| Arachnida | Araneae | Caponiidae | | jovem | | X | P |
| Arachnida | Araneae | Corinnidae | | jovem | | 1 | E |
| Arachnida | Araneae | Ochyroceratidae | | jovem | | X | E |
| Arachnida | Araneae | Oonopidae | | jovem | X | | E |
| Arachnida | Araneae | Pholcidae | *Mesabolivar* | *aurantiacus* | X | X | P |
| Arachnida | Araneae | Prodidomidae | | jovem | | X | E |
| Arachnida | Araneae | Salticidae | | jovem | X | | E |
| Arachnida | Araneae | Segestriidae | | jovem | | X | E |
| Arachnida | Araneae | Theridiidae | | jovem | | X | E |
| Arachnida | Araneae | Theridiosomatidae | | jovem | X | | P |
| Arachnida | Opiliones | Cosmetidae | *Roquettea* | *singularis* | 1 | | E |
| Arachnida | Opiliones | Stygnidae | *Protimesius* | *gracilis* | 1 | | E |
| Arachnida | Pseudoscorpiones | Chthoniidae | *Pseudochthonius* | sp.1 | X | | E |
| Chilopoda | Scutigeromorpha | Pselliodidae | *Sphendononema* | jovem | | 3 | P |
| Entognatha | Collembola | Paronellidae | | sp.1 | X | X | E/P |
| Insecta | Blattodea | | | jovem | 1 | 3 | E |
| Insecta | Coleoptera | Carabidae | | sp.3 | X | | E/P |
| Insecta | Diptera | Cecidomyiidae | Cecidomyiinae | sp. | | X | E |
| Insecta | Diptera | Chironomidae | | sp. | X | | P |
| Insecta | Diptera | Psychodidae | Phlebotominae | sp. | X | X | P |
| Insecta | Heteroptera | Reduviidae | Reduviinae | sp. | 1 | | E |
| Insecta | Homoptera | Cixiidae | | jovem | X | | E |
| Insecta | Hymenoptera | Formicidae | *Crematogaster* | sp.1 | X | | E |
| Insecta | Hymenoptera | Formicidae | *Dolichoderus* | aff. *bispinosus* | X | X | E |
| Insecta | Hymenoptera | Formicidae | *Odontomachus* | sp.1 | X | | E |
| Insecta | Hymenoptera | Formicidae | *Pheidole* | sp.3 | | X | E |
| Insecta | Isoptera | | | sp. | | X | E |
| Insecta | Lepidoptera | Noctuoidea | | sp.6 | | 1 | E |
| Insecta | Orthoptera | Phalangopsidae | *Paraclodes* | sp. | 10 | 18 | E |
| Amphibia | Anura | Strabomantidae | *Pristimantis* | cf. *fenestratus* | 4 | 1 | E/P |
